# TNF-α and neuropathic pain - a review

**DOI:** 10.1186/1742-2094-7-27

**Published:** 2010-04-16

**Authors:** Lawrence Leung, Catherine M Cahill

**Affiliations:** 1 Centre for Neurosciences Studies, 18, Stuart Street, Queen's University, Kingston, Ontario K7L 3N6, Canada; 2 Centre of Studies in Primary Care, Queen's University, Kingston, Ontario K7L 5E9, Canada; 3 Department of Family Medicine, 220 Bagot Street, Queen's University, Kingston, Ontario K7L 5E9, Canada; 4 Department of Pharmacology & Toxicology, 18, Stuart Street, Queen's University, Kingston, Ontario K7L 3N6, Canada; 5 Department of Anesthesiology, 76 Stuart Street, Queen's University, Kingston, Ontario K7L 2V7, Canada

## Abstract

Tumor necrosis factor alpha (TNF-α) was discovered more than a century ago, and its known roles have extended from within the immune system to include a neuro-inflammatory domain in the nervous system. Neuropathic pain is a recognized type of pathological pain where nociceptive responses persist beyond the resolution of damage to the nerve or its surrounding tissue. Very often, neuropathic pain is disproportionately enhanced in intensity (hyperalgesia) or altered in modality (hyperpathia or allodynia) in relation to the stimuli. At time of this writing, there is as yet no common consensus about the etiology of neuropathic pain - possible mechanisms can be categorized into peripheral sensitization and central sensitization of the nervous system in response to the nociceptive stimuli. Animal models of neuropathic pain based on various types of nerve injuries (peripheral versus spinal nerve, ligation versus chronic constrictive injury) have persistently implicated a pivotal role for TNF-α at both peripheral and central levels of sensitization. Despite a lack of success in clinical trials of anti-TNF-α therapy in alleviating the sciatic type of neuropathic pain, the intricate link of TNF-α with other neuro-inflammatory signaling systems (e.g., chemokines and p38 MAPK) has indeed inspired a systems approach perspective for future drug development in treating neuropathic pain.

## Introduction

Despite intense research over the last 30 years, debate is still ongoing regarding the nature of neuropathic pain, including controversy as to whether such pain is peripheral or central in origin, and as to whether its etiology is inflammatory or non-inflammatory. Increasing evidence has provided better understanding of the roles of both immune and pro-inflammatory mediators (e.g., the interleukins, TNF-α, complement components, ATP and the chemokines) in the mechanisms of both peripheral and central neuropathic pain [1-4]. This review will concentrate on current knowledge and experimental models regarding the role of TNF-α, among other cytokines, in neuropathic pain; with an appraisal of available potential therapeutic targets related to TNF-α and directions for future developments in this area.

### Neuropathic pain as an example of an inflammatory pain model

Neuropathic pain is characterized by disproportionate hypersensitivity to stimuli (hyperalgesia), abnormal pins-and-needles or electric-shock-like sensations (hyperpathia) and, finally, nociceptive responses to non-noxious stimuli (allodynia). It is a pathological type of pain that persists despite resolution of the inciting damage to the nerve and the surrounding tissues. From a behavioral standpoint, nociception is an adaptive tool for better survival, while neuropathic pain is considered maladaptive. The prevalence of neuropathic pain ranges from 1% in UK [5] to 1.5% in the US [6] to 17.9% in Canada [7]. Weir Mitchell [8] is often credited with the first descriptive account of neuropathic pain from nerve injuries seen in the US Civil War, using terms that range from "burning", "mustard red hot", "red-hot file rasping the skin" to "with intensity ranging from most trivial burning to a state of torture". Clinically, the top three most common types of neuropathic pain are post-herpetic neuralgia, trigeminal neuralgia and diabetic neuropathy [9]. Neuropathic pain is among the most difficult types of chronic pain to treat, which not only significantly impairs patients' quality of life [10] but also adds to the burden of direct and indirect medical cost for our society [10,11]. Conceptually, neuropathic pain consequent to peripheral nerve injury results from an increased excitability of the neurons as a result of sensitization. The debate is still on-going as to whether this sensitization occurs in the peripheral or central compartments of the nervous system, or both. Experimentally, various animal models of peripheral neuropathic pain have been developed: chronic constriction injury (CCI) of the sciatic nerve with loose ligatures [12-15]; partial sciatic nerve injury with tight ligatures [15-17]; total sciatic nerve ligation [15,18]; sciatic nerve transaction [19-21] and axotomy of lumbar roots entering the sciatic nerve [22,23]. Despite the various degrees and modes of nerve damage in these models, there is a common sequel--post-injury inflammatory changes leading to mast cell degranulation [24], and recruitment of both macrophages [25] and polymorphonuclear neutrophils [26]. However, in CCI models thermal hyperalgesia still occurs when ligatures are loosely placed around the sciatic nerve without actual mechanical damage [27]. This finding supports the hypothesis that it is the inflammatory microenvironment [28] and the release of mediators [29], rather than the nerve injury *per se*, that is pivotal for the development of neuropathic pain. Clatworthy et al. [30] further demonstrated that suppression of the inflammatory response with dexamethasone reduces thermal hyperalgesia, while enhancing the inflammatory response using Freud's adjuvant was seen to aggravate the level of pain hypersensitivity. His work set the stage for continuing research on immune and pro-inflammatory mediators in neuropathic pain over the next two decades. An updated list of such mediators, by no means exhaustive, includes the eicosanoids [31-34], bradykinins [35,36], serotonin [37-39], ATP/ADP [40-42], neurotrophins [43-46], cytokines [47-52], chemokines [53,54], and reactive oxygen species [21,55,56]. These mediators are not exclusive to cells of immune/inflammatory origin, but are also produced by Schwann cells [57-59] and spinal glial cells [42,60-63], thereby potentially mediating the mechanisms of neuropathic pain.

### Cytokines in neuropathic pain

Cytokines are low molecular weight glycoproteins that are secreted mainly, but not exclusively, by immunological cells such as T-cells, macrophages and neutrophils. Other cells that secrete cytokines include keratinocytes and dendritic cells of the skin [64] and Schwann cells and glial cells of the central nervous system [65,66]. They act as intercellular mediators regulating the functions and differentiation of neighboring cells and are produced in response to disease, inflammation, or tissue damage. Cytokine synthesis is prompt and their actions are often localized with a relatively short half-life. This distinguishes them from hormones which are constantly produced with longer-lasting and more distant effects. The first cytokine was discovered by Beeson in 1948 [67] as a pyrogenic compound extracted from ploymorphonuclear leucocytes, later known as IL-1β. Since then, many other cytokines have been discovered, and these fall into five main categories: interleukins, interferons, tumor necrosis factors, growth factors and chemokines. Together, these factors contribute to the pathogenesis of neuropathic pain [47,68]. In particular, tumor necrosis factor alpha (TNF-α) [69,70], interleukin-1 (IL-1) [47,71,72] and interleukin-6 (IL-6) [49,73] have been associated with the development of neuropathic pain in various animal models [74]. In this review, we shall limit our scope to TNF-α.

### Tumor necrosis factor alpha (TNF-α): a neuropathic pain-related cytokine

In 1891, the success story of William Coley in using supernatant extract of heat-killed mixtures of *Streptococcus pyogenes *and *Serratia marcescens *bacteria to treat tumors may in fact be the first discovery of tumor necrosis factor [75]. It was not until 1975 that an endotoxin-like substance was described in activated macrophages with tumor-regression activity and was given the name of tumor necrosis factor alpha, TNF-α [76]. TNF-α belongs to a superfamily of ligand/receptor proteins called the tumor necrosis factor/tumor necrosis factor receptor superfamily proteins (TNF/TNFR SFP). TNF-α possess a trimeric symmetry with a structural motif called the TNF homology domain (THD), which is shared with all other members of the TNF proteins. This THD binds to the cysteine-rich domains (CRDs) of the TNF receptors (TNFRs), and variations of these CRDs lead to heterogeneity of the TNFRs [77]. TNFRs are either constitutively expressed (TNFR1, p55-R) or inducible (TNFR2, p75-R) [78]. In the context of neuropathic pain, using the standard model of chronic constriction injury (CCI) of sciatic nerve in rats, TNF-α has been detected at the injury site and shows temporal up-regulation [79-81]; here TNF-α is located mainly in macrophages [82] and Schwann cells [70,83] by immuno-reactive staining. Similarly, there is local up-regulation of both TNFR1 and TNFR2 as injured neurons undergo Wallerian degeneration, albeit at differential rates [84]. Similar results are found in humans, where nerve biopsies from patients with painful neuropathy show higher levels of TNF-α expression, especially in Schwann cells [85]. Intra-sciatic injection of TNF-α in rats reproduces pain hypersensitivity that is similar to that of neuropathic pain in humans [69,86], and this is reversible with neutralizing antibodies to TNFR [86], in particular TNFR1 [50]. TNF-α enhances the tetrodotoxin-resistant (TTX-R) Na^+ ^current in cultured DRG cells from wild-type but not from TNFR1-knockout mice, and such current is abolished by a p38-MAPK inhibitor; implying that TNF acts via TNFR1 and activates TTX-R Na^+ ^channels via the p38 MAPK system [87]. Further studies using TNFR1/TNFR2 knock-out mice have suggested a neurotoxic role for TNFR1 versus a neuroprotective role of TNFR2 [88]. However, there is still debate regarding the relative roles of TNFR1 and TNFR2 in chronic pain: in mice with tumor-induced thermal hyperalgesia, deletion of the TNFR2 gene reduces the painful response hence signifying a role for TNFR2 [89]; whilst in rats with spinal root injury, TNFR1 elicits excitatory responses in DRG of adjacent uninjured roots and TNFR2 excites DRG neurons from injured roots [90]. In the inflammatory models of carrageenan-induced and zymosan-induced pleurisy in rat models, TNF-α has been found to have a lead role in activating a cascade of other cytokines, notably IL-1β, IL-6 and IL-8 [91]. A similar local cascade has been demonstrated in a model of neuropathic pain following nerve injury [83].

### The role of TNF-α in peripheral mechanisms of neuropathic pain

TNF-α plays a role in the peripheral mediation of neuropathic pain. Clinically, HIV therapy and chemotherapy produce peripheral neuropathy with massive release of TNF-α in serum [92] and TNF-α used as a clinical anti-cancer treatment leads to peripheral neuropathy [93]. Traditional CCI of sciatic nerve in rats results in raised levels of TNF immunoreactivity in dorsal root ganglia (DRG) of both injured and uninjured ipsilateral adjacent afferents [94], as well as of contralateral uninjured counterparts [95], which can only be partly explained by retrograde axonal transport [96]. There is also a corresponding up-regulation of TNFR1 and TNFR2 in both nerve and DRG [97], with a temporal pattern of increased TNF mRNA expression, first in sciatic nerve, and then in DRG [98]. When nucleus pulposus extract of coccxygeal intervertebral disc is applied to lumbar DRG of rats, neuropathic pain is induced but is abolished by co-application of TNRF1, implying a direct role of TNF as a local mediator [99]. Exogenous TNF-α injected into DRG of CCI roots is transported both anterograde to the site of injury and retrograde into the dorsal horn [100], precipitating allodynia in both the ligated and adjacent uninjured nerves [101]. TNF-α is known to lead to apoptosis via TNFR1 [102,103] and the caspase signaling pathway [103]. Caspase inhibitors can attenuate peripheral neuropathy experimentally induced by HIV therapy or chemotherapy in rats [104]. A recent study compared crush injury of L5 spinal nerve (distal to DRG) with L5 nerve roots (proximal to DRG) in rats and found that distal crush injury resulted in more neuronal apoptosis and enhanced TNF-α expression and caspase levels, correlating with higher neuropathic pain [105], lending more support to a TNF-α-apoptosis-caspase signaling paradigm for peripheral neuropathic pain. In addition to enhancing TTX-R Na^+ ^channels in nociceptive DRG neurons [87], TNF-α can also increase membrane K^+ ^ion conductance in a non-voltage-gated fashion [106] leading to overall neuronal hyper-excitability and hence leading to neuropathic pain.

### The role of TNF-α and glia in central mechanisms of neuropathic pain

In late 1990s, TNF-α was proposed to be one pro-inflammatory cytokine with a pivotal role in the "immune-to-brain" pathway of communication for pain, and in models of sickness response in general [51,107]. In classic CCI models in rats, increased levels of TNF-α are found in hippocampus [108,109], locus coeruleus [109,110] and red nucleus [111] of brain. Recent data have suggested that TNF-α mediates central mechanisms of neuropathic pain through glial systems. In the central nervous system, glial cells outnumber neurons by as much as 50-fold, and include three relevant types: astrocytes, oligodendrocytes and microglia. Oligodendrocytes not only provide the myelin sheaths that insulate the neurons, they also contribute to the actual expansion of neuronal caliber and reorganization of neurofilaments [112]. Astrocytes are the most abundant glial cells and possess the most diverse functions: they can modulate synaptic functions by forming a tripartite synapse with pre-synaptic, post-synaptic and extra-synaptic astrocytic contacts with up to 10,000 other neurons [113,114] using glutamate and adenosine as neurotransmitters [115]. It has been suggested that spinal astrocytes may play a role in sensitization of chronic pain via activation of the p38-MAPK system [116,117], and may even synapse with microglia, with pre-synaptic neuronal processes and with post-synaptic neuronal structures to form a tetrapartite configuration [118]. Astrocytes also regulate maturation of neurons and synpatogenesis, hence playing a pivotal role in modulation of neural plasticity [119]. Microglia constitute 15-20% of the total glial population and serve as an immune invigilator for the central nervous system. They originate from tmesodermal precursor cells of hemopoietic lineage. In response to nerve injury and inflammation, microglia transform into macrophage-like cells [120] that express major histocompatibility complex antigens and secrete pro-inflammatory cytokines, including TNF-α, IL-1 and IL-6 [121,122]; CCL2 and CX3CL1 [53,123], and ATP, which mediate their effects via the p38-mitogen-activated protein kinase (p38-MAPK) system [41,124,125].

Back in 1991, it was shown that classic CCI leads to hypertrophy of astroglia in the dorsal horn of spinal cord as reflected by increased immunostaining of glial fibrillary acidic protein [126]. Since then, other subcutaneous and intraperitoneal inflammatory pain models [127] have also been shown to induce glial activation. In newborn rats, where microglia are immature, intrathecal lipopolysaccharide (LPS) fails to evoke the allodynia response that is invariably seen in adult rats [62], suggesting a necessary role for functional microglia in the pathogenesis of neuropathic pain. Along with various other mediators, TNF-α has been shown to be present on the surfaces of astrocytes by immunofluorescence staining, where TNF-α auto-stimulates its own production via G-protein coupled receptor (CXCR4) and TNF-α converting enzyme. The result is a cascade of events leading typically to production of IL-1, IL-6, nitric oxide and ATP [121,128], all of which contribute to enhanced neuronal activity leading to pathological pain. Wei *et al *[129] demonstrated increased levels of TNF-α and IL-1β in the rostral ventromedial medulla (RVM) of rats after CCI of the infraorbital nerve, with a corresponding enhancement of phosphorylation of the NR1 subunit of NMDA receptors, which is thought to be coupled to the receptors for both TNF-α and IL-1β. Injection of TNF-α and IL-1β into RVM increases NR1 phosphorylation of NMDA receptors and produces hyperalgesia, which is reversed by an NMDA antagonist. Wei's work sparked off research into NMDA receptors as a possible target for treating neuropathic pain; unfortunately, progress has been discouraged by the ubiquitous expression of NMDA receptors in the human central nervous system, which renders NMDA receptor blockade for analgesia an impossible task without concomitant alterations in cognition, memory and learning.

### TNF-α, neural plasticity and neuropathic pain

Originally identified in hippocampus as a substrate for memory storage and learning, the synaptic mechanisms of long term potentiation (LTP) in glutamergic neurons [130] have since been demonstrated as well in other parts of the central nervous system; in particular, in the dorsal horn of the spinal cord, where they may lead to abnormal nociception and neuropathic pain [131,132]. Normal nociceptive signals are conveyed by both Aδ and C-fibers; of which the latter make synapses with second-order neurons in the spinal dorsal horn. The LTP phenomenon has been well characterised in C-fibers of rat dorsal horn with tetanic stimulation [133,134] and also with acute nerve injury [135]. High-frequency stimulation leads to an LTP pattern of cutaneous allodynia and hyperalgesia in humans [136] with a typical early LTP time course [137]. As the signalling mechanism of LTP unfolds, TNF-α is found to play an important role. Endogenous glial TNF-α can modulate synaptic plasticity by increasing the expression of AMPA receptors in cultured rat hippocampal slices [138] for homeostatic regulation of synaptic strength in an activity-dependent fashion [139]. However, TNF-α given at non-physiological levels often inhibits LTP in similar models of cultured rat hippocampus [140,141]. As regards to C-fibers in the spinal dorsal horn, exogenous TNF-α produces LTP in C-fiber evoked field potentials only in the presence of nerve injury, and this LTP is blocked by inhibitors of NF-kappa B, JNK and p38-MAPK [142]. In the absence of nerve injury, TNF-α can neutralise the action of src-family kinase inhibitors by restoring LTP in C-fiber evoked potentials as normally induced by high-frequency stimulation (HFS).

### TNF-α, ATP and p38-MAPK

Since the 1950s, release of ATP has been detected from nerve endings [143,144] and a role for ATP in nociception was implicated when it was shown to induce pain in human blister bases [145]. ATP excites cutaneous afferent neurons of animal models in a fashion similar to that of other neurotransmitters like 5-HT and acetylcholine [146], and can act proximally to excite DRG neurons [147]. Around the same time, Burnstock and his colleagues [148,149] first characterized purinergic receptors into P1 (sensitive to adenosine, ADO), P2X, and P2Y receptors (sensitive to ATP and ADP). Molecular cloning studies have identified four sub-types of P1 (A1, A2A, A2B, and A3), seven sub-types of P2X (P2X_1 _to P2X_7_) and 8 sub-types of P2Y receptors (P2Y_1_, P2Y_2_, P2Y_4_, P2Y_6_, P2Y_11_, P2Y_12_, P2Y_13_, P2Y_14_) [150]. Each subtype has a different distribution in neuronal and glial cells, interacting with each other in an intricate manner. In terms of signaling functions, P1 and P2Y receptors are G-protein coupled receptors, while P2X receptors are ligand-gated ion channels [151]. Within the context of neuropathic pain, P2X_3_, P2X_4 _and P2X_7 _receptors are thought to play a role; and in particular, P2X_3 _may act via the TTX-R voltage-gated sodium channel Na_v _1.9 [152].

Earlier studies using nerve injury models in rats revealed either an increase [153] or decrease [154] of P2X_3 _immuno-reactivity of the DRG neurons, depending on the type of nerve injury. When expression of P2X_3 _receptors in DRG is reduced using anti-sense oligonucleotides [155] or siRNA [156], development of mechanical hyperalgesia is mitigated after classic CCI. Furthermore, administration of anti-sense oligonucleotides to knock down P2X_3 _receptors can reverse established neuropathic pain that re-emerges after cessation of the anti-sense treatment [157], suggesting a dynamic modulatory role of P2X_3 _receptors. Following a similar approach, Tsuda *et al *[158] demonstrated an increase in P2X_4 _receptor expression after chronic nerve injury, and showed that both pharmacological blockade and anti-sense oligonucleotide treatment abrogates the development of mechanical allodynia. Later studies have suggested that P2X_4 _receptor stimulation leads to secretion of brain-derived neurotrophic factor (BDNF) in spinal microglia, and that this BDNF is involved in mediating neuropathic pain [40,159,160], possibly via activation of the p38-MAPK system [161]. P2X_7 _receptors are associated with TNF-α production in microglia through the p38-MAPK system [162,163], as an inhibitor of MAPK system will suppress production of TNF-α mRNA and an inhibitor of p38 will prevent nucleocytoplamic transport of TNF-α mRNA [162]. Independent of ATP, the p38-MAPK system seems to be essential for the action of TNF-α via TTX-R Na^+ ^channels [87]. As an entity itself, microglial p38-MAPK has been implicated in the pathogenesis of neuropathic pain in studies using various in vivo models of peripheral nerve [164,165] and spinal cord injury [166,167]. For example, spinal nerve ligation in rats leads to allodynia with concomitant rises in TNF-α and p38 phosphorylation; treatment with inhibitors of either TNF-α or p38 results in reduction of allodynia and, finally, TNF-α blockade can in turn suppress p38 activation [168]. Studies using HSV-mediated gene transfer in nerve injury animal models have shown induced expression of soluble p55 TNFR (sTNFR2) in DRG neurons, resulting in decreased phosphorylation of p38 and reduced allodynia, again suggesting a causal link between TNF-α and the p38-MAPK system.

### TNF-α as potential drug target for chronic pain--the possibilities

Due to the unique trimeric structure shared between the TNF ligand and the TNF receptor (both belonging to the TNF/TNFR SPF), the transmembrane portion of TNF molecule (mTNF), besides being a ligand, is capable of acting as a receptor for a soluble form of TNF (sTNF) in a "reverse-signaling" manner [169], which then inhibits phosphorylation of p38 and hence expression of TNF protein. This unique phenomenon makes it possible to use gene therapy with a herpes simplex virus vector carrying a p55 sTNFR gene to transfect DRG neurons of rats [170]. As a result, over-expressed p55 sTNFR (sTNFR2) binds to the mTNF of DRG and down-regulates overall production of TNF by reverse signaling, significantly reducing the allodynia and hyperalgesia responses to CCI [170,171]. Following a similar logic, a fusion protein (ELP-sTNFR2) has been developed wherein a soluble form of TNFR2 (sTNFR2) is conjugated to a temperature-sensitive elastin-like polypeptide (ELP), which can be thermally triggered to form a deposit around the peri-neural site of injection [172]. This fusion protein has been reported to be able to mitigate levels of TNF-α in DRG of injured nerve in rat models [173]. Indeed, many studies have demonstrated that local or spinal administration of agents that antagonize TNF-α will attenuate pain behaviors in neuropathic animal models [174-177]. Mechanical allodynia in the rat model of central neuropathic pain due to T13 spinal cord hemisection is attenuated by immediate, but not delayed, intrathecal administration of etanercept (a fusion protein blocker of TNF-α) at 1-4 weeks post spinal cord injury [178]. Propentofylline is a methylxanthine that inhibits lipopolysaccharide (LPS)-induced release of both TNF-α and IL-1β in a dose-dependent manner in glial cultures [179] and abates allodynia in rat spinal nerve transection models by modulating glial activation [180,181]. Propentofylline was initially evaluated for treating dementia [182], but was eventually withdrawn from further clinical studies due to patent issues [183], and its efficacy in animal neuropathic pain models has yet to be tested in humans. Thalidomide, once banned in 1963 due to its teratogenicity, is now regaining favor in neuropathic pain research due to its ability to cross the BBB and its inhibitory effects on TNF-α (in vitro and in-vivo) and on IL-1/IL-6 (in-vitro only) [184,185]. In the rat model of CCI, systemic thalidomide reduces the hyperalgesia response coincident with reductions in TNF-α levels, unchanged levels of IL-1/IL-6 and increased levels of IL-10 [186,187]. Clinically, there have been sporadic reports of success in using thalidomide to treat complex regional pain syndromes [188]. However, the balance of thalidomide's efficacy versus safety in treating in chronic and neuropathic pain needs further clinical study [189], especially in view of its paradoxical neurotoxicity [189,190]. Methotrexate is a well-known drug for treating cancer that is derived from glutamic acid. It is capable of crossing the BBB [191] and has anti-rheumatoid and anti-inflammatory actions through its inhibition of production of TNF-α via adenosine nucleotides [192,193] and its ability to antagonize the actions of IL-1 [194]. Intrathecal administration of methotrexate reduces classic CCI-induced allodynia in rats [195] but its value in treating neuropathic pain is severely offset by its propensity *per se *to induce astrocytic proliferation [196] and hence neurotoxicity [197,198].

The role of TNF-α in chronic pain seems irrefutable in view of abundant data from various neuropathic animal models, and with the actual isolation of TNF-α from neuropathic nerves [85] and perineural fat from radiculopathic nerve roots [199] in humans. An initial pilot study using subcutaneous etanercept to treat patients admitted to the hospital with acute severe sciatica showed improved pain scores [200]. Similarly, an open-label study with infliximab (an antibody to TNF-α) revealed promising results [201]. Subsequent randomized controlled trials failed to support the benefits of systemic anti-TNF-α treatment [202-205], but a recent report did show positive benefits of epidurally administered etanercept in the treatment of sciatica [206]. To date we are unaware of any randomized controlled clinical trials of infliximab or etanercept in treating other types of neuropathic pain. AV411(ibudilast), a trial drug that was originally developed as a non-selective phosphodiesterase inhibitor for treating bronchial asthma, has been studied in phase I and phase 2a clinical trials in the US and in Australia for treatment of diabetic neuropathic pain [207], based on findings that AV411 also suppresses glial cell activation and reduces the production of pro-inflammatory cytokines (IL-1β, TNF-α, IL-6) in rat neuropathic pain model [208].

### Perspective on future studies

**Figure 1:**
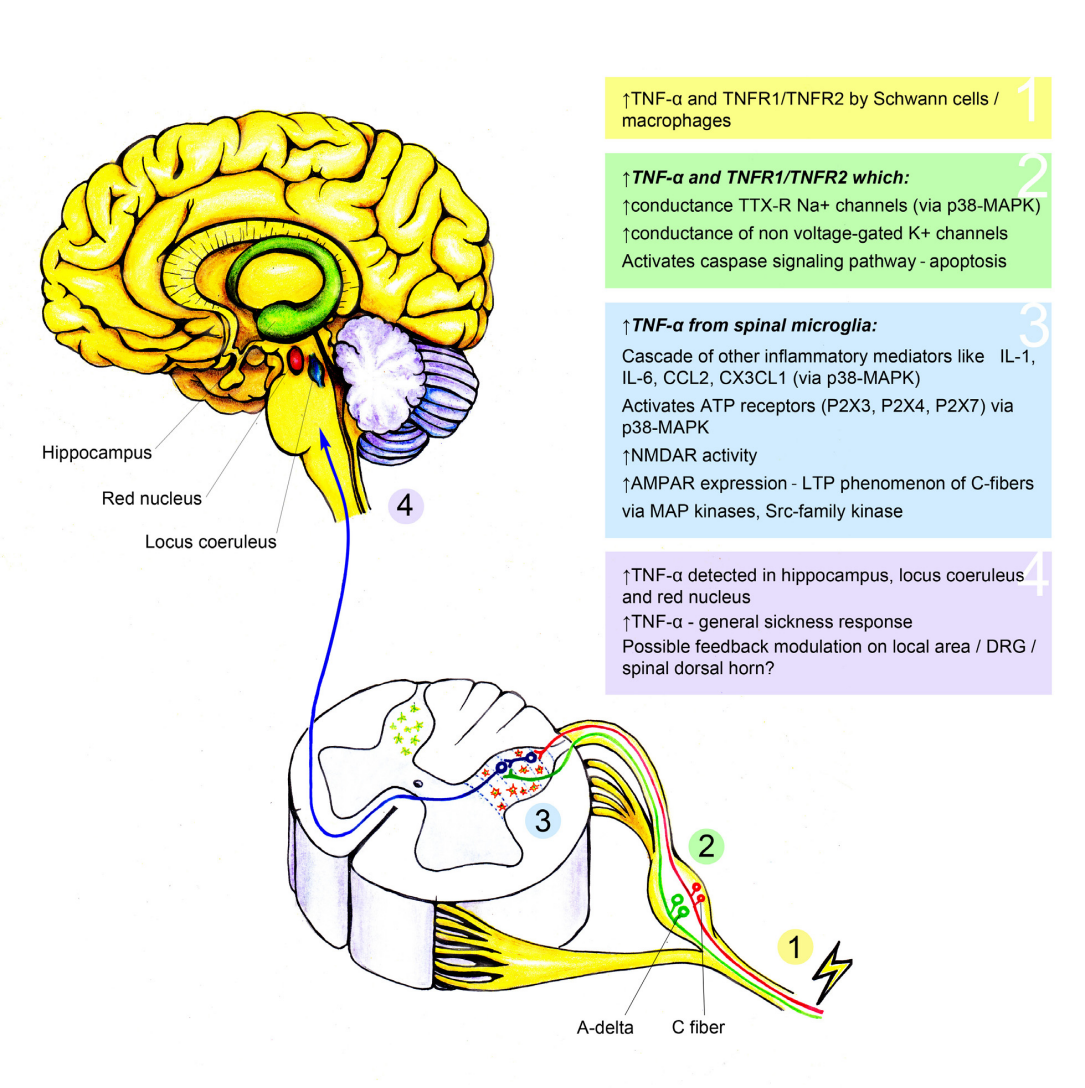
**The roles of TNF-α as recognized at different levels of the nervous system in neuropathic pain induced by nerve injury: (1) at site of nerve injury; (2) at dorsal root ganglion; (3) at dorsal horn of the spinal cord; and (4) at the brain and higher centres**.

TNF-α is undoubtedly a titan in the research of neuropathic pain, and is by no means the only one in the arena. It is a pivotal member of the cytokine mediator system that is intrinsic to the pathogenesis of neuropathic pain both at peripheral and central levels (See Fig 1). Together with other mediators like interleukins, nerve growth factors, chemokines and interferons, it forms a network that interacts with downstream signaling mechanisms like the NMDA, ATP and MAPK systems. We now know that removing TNF-α from the picture will not abolish neuropathic pain as has already been demonstrated by the failure of TNF-α antagonists in clinical trials for sciatica [202-205]. Emerging data have guided research towards a collective role for glia-derived mediators and their coupled signaling pathways in the modulation of neuropathic pain [122,127,209,210]. The paradigm is shifting from a single compound towards a system as a potential target for novel drug development for treating neuropathic pain [211-214]. Examples include the chemokine system [215], the MAPK system[216], and the glial system as a whole [217].

## Abbreviations

5-HT: 5-hydroxytryptamine (Serotonin); ADO: Adenosine; ADP: Adenosine diphosphate; ATP: Adenosine triphosphate; BBB: Blood-brain barrier; BDNF: Brain-derived neurotrophic factor; CCI: Chronic constrictive injury; CCL2: Chemokine (C-C motif) ligand-2; CX3CL1: Chemokine (C-X3-C motif) ligand 1; CXCR4: CXC chemokine receptor-4; DRG: Dorsal root ganglion; CRDs: Cysteine rich domains; ELP: Elastin-like polypeptide; ERK: Extracellular signal-regulated kinases; HFS: High Frequency stimulation; HSV: Herpes simplex virus; IL-1: Interleukin-1; IL-1β: Interleukin-1 beta; IL-6: Interleukin-6; IL-8: Interleukin-8; IL-10: Interleukin-10; JNK: c-Jun N-terminal kinases; LPS: Lipopolysaccaride; LTP: Long Term Potentiation; MAPK: Mitogen activated protein kinase; NMDA: N-methyl-D-aspartic acid; NSAIDs: Non-steroidal anti-inflammatory drugs; siRNA: Small interfering RNA; RVM: Rostral ventromedial medulla; THD: TNF homology domain; mTNF: Transmembrane portion of TNF; sTNF: Soluble form of TNF; TNF-α: Tumor necrosis factor alpha; TNFR: Tumor necrosis factor receptor; sTNFR2: Soluble p55 TNF receptor; TNFR SFR: Tumor necrosis factor receptor super family receptor; TTX-R: Tetrodotoxin resistant.

## Competing interests

The authors declare that they have no competing interests.

## Authors' contributions

LL wrote the manuscript, CMC provided comments and proof-reading. Both authors have read and approved of the final version of the manuscript.
